# Growth factor expression is enhanced, and extracellular matrix proteins are depressed in healing skin wounds in septic patients compared with healthy controls

**DOI:** 10.1111/apm.13175

**Published:** 2022-01-23

**Authors:** Henna Jaurila, Marjo Koskela, Vesa Koivukangas, Fiia Gäddnäs, Tuula Salo, Tero I. Ala‐Kokko

**Affiliations:** ^1^ Research Group of Surgery, Anesthesia and Intensive Care Oulu University Hospital Medical Research Center Oulu University of Oulu Oulu Finland; ^2^ Cancer and Translational Medicine Research Unit Faculty of Medicine Medical Research Center Oulu University of Oulu Oulu Finland; ^3^ Research Group of Oral Health Sciences Oulu University Hospital Medical Research Center Oulu University of Oulu Oulu Finland

**Keywords:** Sepsis, skin wound, immunohistochemistry, ECM, growth factor

## Abstract

Sepsis manifests as a dysregulated immune response to infection, damaging organs. Skin has a critical role in protecting the body. In sepsis, skin wound healing is impaired. The mechanisms behind it have been poorly studied. In this study, suction blister wounds were induced on intact abdominal skin in 15 septic patients. A single blister wound was biopsied from each patient and from 10 healthy controls. Immunohistochemical staining of growth factors and extracellular matrix (ECM) proteins was performed. Significance (*p* < 0.05) of the differences was calculated. The following growth factors were overexpressed in the skin of septic patients compared with healthy controls: epithelial growth factor (intact epithelium p = 0.007, migrating epithelium p = 0.038), vascular epithelial growth factor (intact epithelium p < 0.001, migrating epithelium p = 0.011) and transforming growth factor beta (migrating epithelium p = 0.002). The expression of syndecan‐1 was upregulated in the skin of septic patients compared with healthy controls (intact epithelium p = 0.048, migrating epithelium p = 0.028). The following ECM proteins had lower expression in the epithelium in septic patients than in healthy controls: tenascin‐C (migrating epithelium p = 0.03) and laminin‐332 (intact epithelium p = 0.036). In sepsis, growth factor and syndecan expression was enhanced, while ECM and basement membrane proteins were mostly depressed.

Skin is the only organ, which is persistently in direct contact with the environment, making it a major protective barrier. According to the present nomenclature, sepsis is a dysregulated response to infection that can lead to severe tissue damage, multi‐organ failure and death [1]. Critical illness and its care disrupt skin homeostasis, and the skin is prone to infection, blistering, necrosis, pressure ulcers, fascial dehiscence and delayed wound healing [2, 3, 4].

Interaction between extracellular matrix (ECM), epithelial cells and growth factors is fundamental in all phases of wound healing, and abnormalities in the expression of these components may lead to disturbed regeneration of tissues [5]. ECM provides a scaffold for migrating cells and adhesion sites for molecules, thus acting as a temporary reservoir for growth factors before targeting receptors on the cell surface [6]. The ECM, and the basement membrane (BM) as part of it, regulates the skin integrity and regeneration. Cytokines and growth factors are cell‐signalling molecules that stimulate cell growth, migration and proliferation. Keratinocyte proliferation and migration are essential in re‐epithelialization of the wound, and keratinocytes are also an important source of growth factors [7]. In our previous in vitro study, we found out that a keratinocyte cell line treated with sepsis sera had diminished migration, proliferation and viability compared with keratinocytes treated with healthy sera, and one possible reason could be the decreased levels of epithelial growth factor (EGF) [8].

Some studies have confirmed delayed skin wound healing in sepsis in humans [9] and in animal models [3, 10, 11, 12]. In septic rodents, wound re‐epithelialization is delayed [3, 10, 11] and collagen synthesis and deposition are decreased in skin leading to deteriorated wound breaking strength [3, 12, 13, 14, 15]. Neutrophil influx into the secondary inflammatory site (skin wound blisters) is reduced in septic patients compared to healthy controls [16]. In our previous human studies, the expression of laminin‐332 and type IV collagen in the BM of intact skin of septic patients has been shown to be diminished [17], as has skin collagen synthesis [18]. Cytokine responses in skin blister fluid in patients with sepsis are different from those with healthy controls [19], and higher matrix metalloproteinase (MMP) levels are associated with more severe organ dysfunction in sepsis [20]. Otherwise, studies covering the alterations in intact and damaged human skin in sepsis are scarce.

In this study, we compared immunohistochemical differences in growth factors, ECM and BM protein expression in injured septic and healthy skin samples using a blister wound model. We hypothesized that ECM protein expression would be depressed and would serve as a marker of dysregulated wound healing in sepsis.

## MATERIAL AND METHODS

### Patients

This study is an extension of earlier studies dealing with healing response in severe sepsis [17, 21]. The study was conducted over 20 months in a mixed‐type intensive care unit (ICU) at the tertiary‐level referral hospital of Oulu University Hospital, Oulu, Finland. The inclusion criteria for the study were diagnosis of severe sepsis according to 2001 international sepsis definitions [22] and age over 18 years. The exclusion criteria were bleeding disorder, chronic renal or hepatic failure, malignancy, immunosuppressive medication not related to sepsis and surgery during the preceding six months. All patients had to enter the study after the diagnosis of severe sepsis had been assigned, but within 48 h after the first sepsis‐related organ dysfunction was found. Informed consent was obtained from all study patients or their surrogate decision‐maker. From the larger patient population of 44, we picked 15 subsequent patients with informed consent. The study protocol (register number 50/2005) was approved by the Regional Ethics Committee of the Northern Ostrobothnia Hospital District, Finland. Patients were treated according to the normal ICU protocol and sepsis guidelines applicable at the time of the study [23]. A wide range of clinical variables was collected daily during the ICU stay, and 30‐day mortalities were recorded. Healthy age‐matched Caucasian volunteers served as controls.

### Skin wounds

Suction blister method was used to separate the epidermis from the dermis within the BM [24]. The method and study protocol have been described in our earlier paper [9]. In brief, the suction device (Dermovac blistering device; Mucel Co., Nummela, Finland) containing five 8mm‐diameter bores was applied on the intact abdominal skin of septic patients upon entering the study and standardized blisters were formed. The blister roofs were removed, and the remaining epithelial wounds were observed. A skin biopsy of a single blister was taken with a biopsy scalpel under local anaesthesia (1% lidocaine) on Day 3/4/5/6/7 counted from the induction of the experiment, one biopsy per person. The remaining wound was closed with stitches, and the sutures were removed after seven days. Blister wounds were induced with the same devices and according to the same principles in healthy controls, and one wound per person was excised on Day 3/4/5/6/7 counted from the beginning of the experiment.

### Immunohistochemistry methods

All fresh skin samples were immediately fixed in 10% phosphate‐buffered formalin after the biopsy and embedded in paraffin. Tissue sections of 4 µm in thickness were prepared. Antibodies against procollagen type I aminoterminal propeptide (PINP), type IV collagen, tenascin‐C, laminin‐332, alpha smooth muscle actin (α‐SMA), syndecan‐1 (or CD138), EGF, vascular epithelial growth factor (VEGF) and transforming growth factor beta (TGF‐β) were used for immunohistochemical staining according to the manufacturer’s instructions. Type, source, dilution and detection kit of each antigen are shown in Table S1. As a positive control, skin was used. As a negative control, phosphate‐buffered saline was used instead of primary antibody in all samples. The distribution of the staining was observed in the basal and suprabasal layers of the epithelium as well as in the BM and dermis. The staining intensity in each layer was estimated with a four‐level scoring (0 = no staining, 1 = weak staining, 2 = moderate staining and 3 = strong staining). We inspected the samples in three sections horizontally: intact skin, migrating edge of the wound and the wound bed itself, if available. The evaluation and scoring of the staining were performed by two authors (HJ and MK) in consensus, followed by calibration with a pathologist (TS). The slides were number‐coded, and thus, could not be identified as belonging to a septic patient or a healthy control.

### Statistical analysis

The statistical analyses were performed using IBM SPSS Statistics (IBM Corp. Released 2017. IBM SPSS Statistics for Windows, Version 25.0. Armonk, NY, USA: IBM Corp). Patient demographics were presented as medians with quartiles when possible. Differences in staining intensity between groups were analysed with cross‐tabulation, and statistical significance of the differences between groups was calculated with Fischer’s exact test. Values were considered significant at p < 0.05. The bar chart illustration was created by OriginPro (Version 9.1, OriginLab Corporation, Northampton, MA, USA).

## Results

### Patients

In this study, we investigated skin blister wounds taken from 15 patients with severe sepsis and 10 healthy controls. Patient demographics are shown in Table 1. The median age of septic patients was 64 (25th to 75th percentile, 58–72) years, and 11 (73%) of them were male. The median age of healthy controls was 64 (59–70) years, and 4 (40%) of them were male. The skin biopsy of the blister wound was taken from septic patients on day three (n = 2), day four (n = 3), day five (n = 2), day six (n = 4) or day seven (n = 4) post wounding, and from healthy controls on day three (n = 2), day four (n = 2), day five (n = 2), day six (n = 2) or day seven (n = 2), one biopsy per person.

**Table 1:** Patient demographics. Data are expressed as medians and 25th to 75th percentiles or as frequencies and percentages

Characteristic	Septic patients
Number of patients	15
Male gender, n (%)	11 (73%)
Age, years	64 (58–72)
30‐day mortality	2 (13%)
Septic shock, n (%)	14 (93%)
Length of stay in ICU, days	8 (4–11)
APACHE II on admission, points	23 (16–27)
SOFA score on admission, points	6 (5–8)
SOFA score maximum, points	8 (7–10)
MODS, n (%)	5 (33%)
MOF, n (%)	10 (67%)
Sepsis‐related cortisone therapy, n (%)	7 (47%)

ICU, intensive care unit; APACHE II, acute physiology and chronical health evaluation II; SOFA, sequential organ failure assessment; MODS, multiple organ dysfunction syndrome; MOF, multiple organ failure.

### Immunohistochemistry

#### EGF

**Fig. 1:**
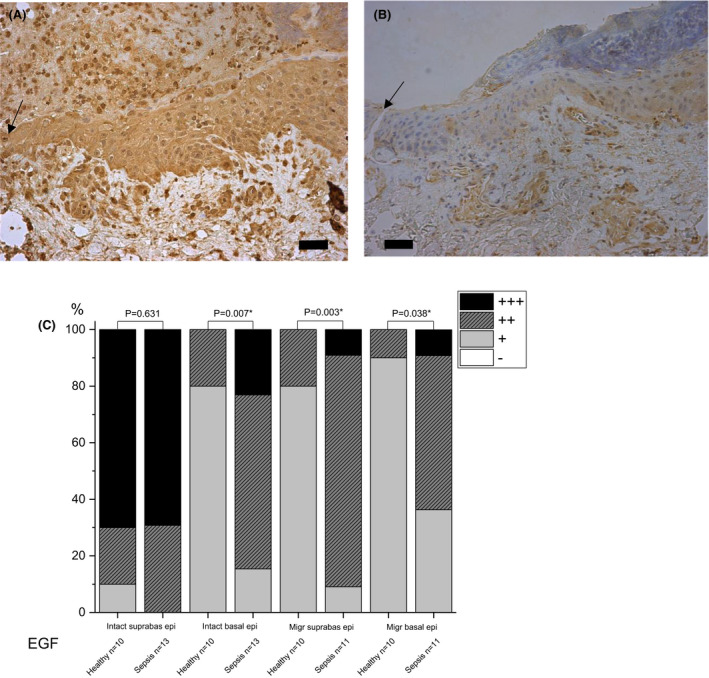
Immunohistochemical staining of epidermal growth factor (EGF) in skin on day 5 post wounding. Samples of septic patients (A) and healthy controls (B) are shown. Migrating wound edge epithelium stained stronger in patient than control samples. Staining intensity is reported as absent (‐), mild (+), moderate (++) or strong (+++), and the percentages are shown (C). The arrow indicates the wound. The bar at the bottom of the figure is equal to 100 µm. Statistical significances between study groups are indicated by brackets and significant p‐values (p < 0.05) are marked with an asterisk.

Staining of the basal layer of the intact epithelium was more intense in sepsis than in controls (moderate staining in sepsis 62% and mild staining in controls 80%, p = 0.007) (Fig. 1). All microscopy snapshots are most representative figures of the samples. Migrating wound edge epithelium was stained stronger in sepsis than in controls (moderate staining of suprabasal layer in sepsis 82% and mild staining in controls 80%, p = 0.003; in basal layer in sepsis moderate staining in 55% and mild in 36%, whereas in controls mild staining in 90%, p = 0.038).

#### VEGF

**Fig. 2:**
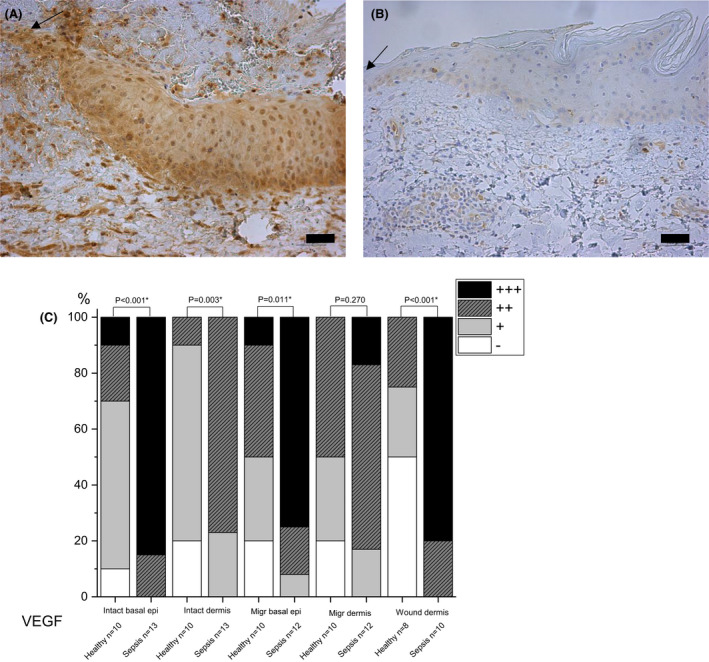
Immunohistochemical staining of vascular epithelial growth factor (VEGF) in skin. Samples of septic patients on day 4 post wounding (A) and healthy controls on day 3 (B) are shown. Septic patients have more intense staining of migrating epithelium than healthy controls. The intensity of staining is reported as absent (‐), mild (+), moderate (++) or strong (+++), and the percentages are shown (C). The arrow indicates the wound. The bar at the bottom of the figure is equal to 100 µm. Statistical significances between study groups are indicated by brackets and significant p‐values (p < 0.05) are marked with an asterisk.

Sepsis patients had stronger staining of VEGF in intact epithelium (in sepsis 85% vs. 10% in controls had strong staining of basal and suprabasal epithelium, p < 0.001 and p = 0.001 respectively) and migrating wound edge epithelium (strong staining of basal epithelium in sepsis 75% vs. 10% in controls and mild staining of suprabasal epithelium 75% vs. 20%, p = 0.011 and p = 0.038 respectively) compared to healthy controls. Sepsis samples also had more intense staining of VEGF in intact dermis (moderate staining in sepsis 77% vs. 10% in controls, p = 0.003) and dermis of the wound site (strong staining in sepsis 80% vs. 0% in controls, p < 0.001) than in healthy controls (Fig. 2). No significant difference was detected in the dermis on the migrating wound edge between sepsis and control groups.

#### TGF‐beta

**Fig. 3:**
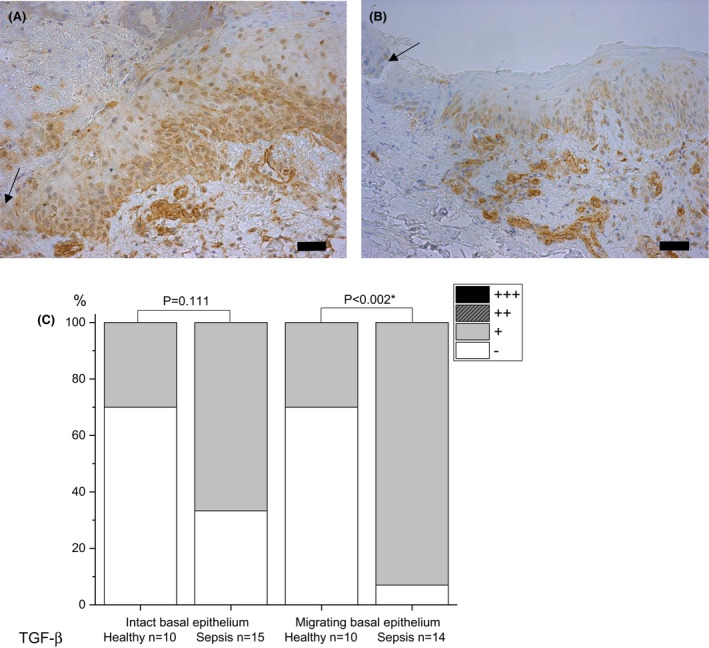
Immunohistochemical staining of transforming growth factor beta (TGF‐β) in skin on day 5 post wounding. Samples of septic patients (A) and healthy controls (B) are presented. The basal layer of migrating wound epithelium is stained more strongly in patient than control samples. Staining intensity is reported as absent (‐), mild (+), moderate (++) or strong (+++), and the percentages are shown (C). The arrow indicates the wound. The bar at the bottom of the figure is equal to 100 µm. Statistical significances between study groups are indicated by brackets and significant p‐values (p < 0.05) are marked with an asterisk.

The basal layer of migrating wound epithelium had mild staining of TGF‐β in septic patients (93%) compared with no staining in healthy controls (70%) (p = 0.002) (Fig. 3). No significant difference emerged in staining of intact basal epithelium between sepsis and control groups.

#### Syndecan‐1

**Fig. 4:**
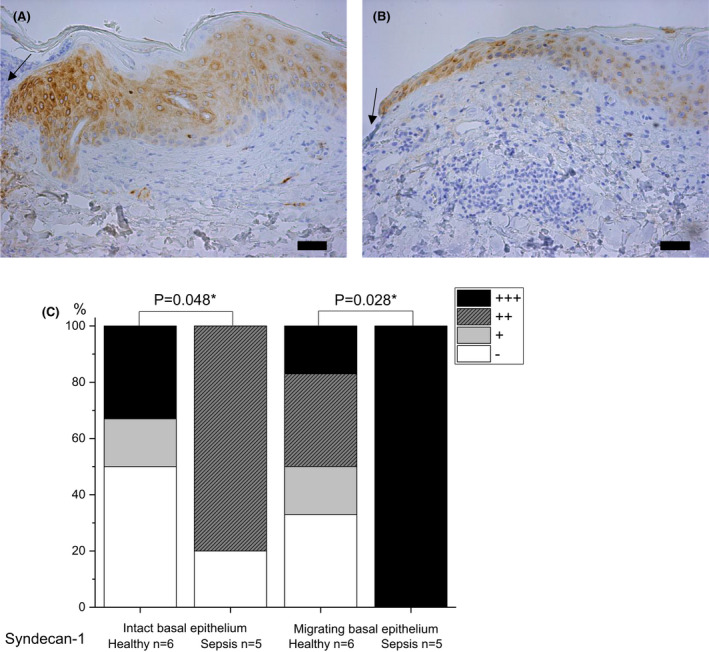
Immunohistochemical staining of syndecan‐1 in skin samples of septic patients on day 4 post wounding (A) and healthy controls on day 3 (B) . Septic patients have more intense staining of migrating epithelium than controls. Staining intensity is reported as absent (‐), mild (+), moderate (++) or strong (+++), and the percentages are shown (C). The arrow indicates the wound. The bar at the bottom of the figure is equal to 100 µm. Statistical significances between study groups are indicated by brackets and significant p‐values (p < 0.05) are marked with an asterisk.

Staining of syndecan‐1 was more intense in all layers of the intact epithelium in septic skin than in healthy skin (moderate staining in 80% of sepsis samples, no staining in 67% and 50% of control samples in suprabasal and basal epithelium; p = 0.026 and p = 0.048 respectively) (Fig. 4), and in the migrating edge of the wound, staining of the epithelium was moderate to strong in sepsis compared with absent to mostly moderate in healthy samples (strong staining in sepsis 100% vs. 17% in control group in suprabasal epithelium, p = 0.015, and 100% vs. 17% in basal epithelium, p = 0.028).

#### Tenascin‐C

**Fig. 5:**
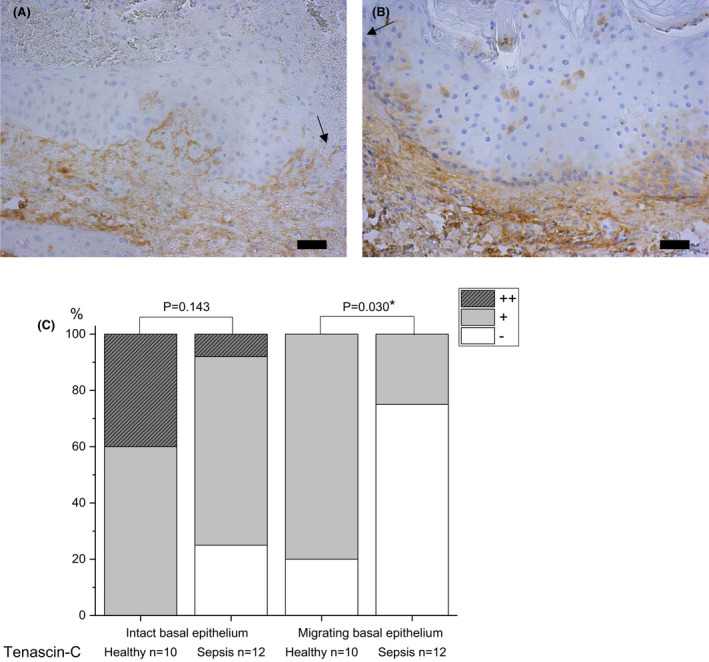
Immunohistochemical staining of tenascin‐C in skin samples of septic patients (A) and healthy controls (B) on day 4 post wounding. The basal layer of migrating epithelium has mostly no staining in septic samples, in contrast to healthy samples. Staining intensity is reported as absent (‐), mild (+), moderate (++) or strong (+++), and the percentages are shown (C). The arrow indicates the wound. The bar at the bottom of the figure is equal to 100 µm. Statistical significances between study groups are indicated by brackets and significant p‐values (p < 0.05) are marked with an asterisk.

In sepsis, the basal layer of migrating wound epithelium had absent staining (75%) in contrast to healthy control samples, which had mild staining (80%) (p = 0.03) (Fig. 5). In sepsis and healthy control samples, there were no significant differences in staining of tenascin‐c between the groups in intact epithelium, and mostly no staining in intact dermis.

#### Laminin‐332

**Fig. 6:**
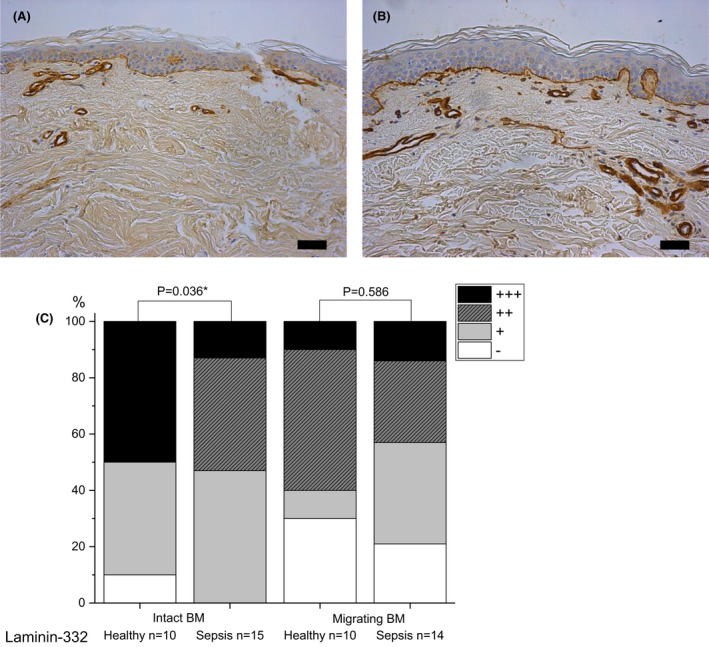
Immunohistochemical staining of laminin‐332 in samples of intact skin on day 6 post wounding. Samples of septic patients (A) and healthy controls (B) are shown. The expression of laminin‐332 in the basement membrane is more intense in healthy than septic samples. Staining intensity is reported as absent (‐), mild (+), moderate (++) or strong (+++), and the percentages are shown (C). The bar at the bottom of the figure is equal to 100 µm. Statistical significances between study groups are indicated by brackets and significant p‐values (p < 0.05) are marked with an asterisk.

In the BM, staining of laminin‐332 was more intense in healthy intact skin (mild in 40% and strong in 50% of wounds) than in septic skin (mild in 47%, moderate in 40% and strong in 13% of wounds) (p = 0.036) (Fig. 6).

#### Alpha‐SMA

**Fig. 7:**
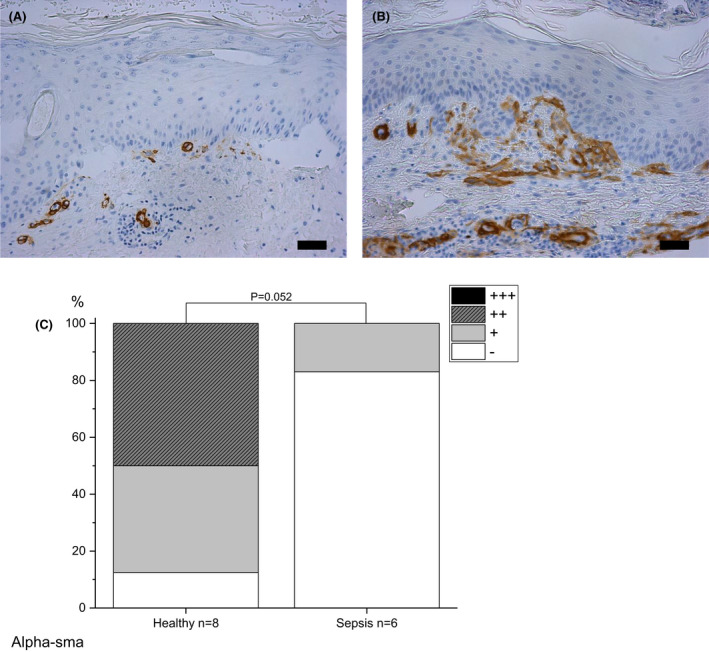
Immunohistochemical staining of alpha smooth muscle actin (α‐SMA) in skin. Samples of septic patients on day 6 post wounding (A) and healthy controls on day 7 (B) are shown. Expression of α‐SMA in septic samples is mostly absent, in contrast to healthy samples. Staining intensity is reported as absent (‐), mild (+), moderate (++) or strong (+++), and the percentages are shown (C). The bar at the bottom of the figure is equal to 100 µm. Statistical significances between study groups are indicated by brackets and significant p‐values (p < 0.05) are marked with an asterisk.

In septic skin, the staining of α‐SMA was mostly absent (83% of cases) in contrast to healthy skin (no staining 12.5%), but this result is slightly over the limit of significance (p = 0.052) (Fig. 7).

#### Type IV collagen

There was mostly mild or no staining in intact or migrating wound edge or wound site skin samples, and no significant differences emerged between sepsis and healthy groups (p = 0.233, p = 0.431, p = 0.692 respectively) (Fig. S1).

#### PINP

In intact dermis, the staining of PINP was mostly mild or moderate (moderate staining in sepsis 58% vs. 50% in controls, mild staining 25% vs. 50%) and mild or absent in dermis of migrating wound edge (mild staining in sepsis 67% vs. 33% in controls, absent staining 33% vs. 56%) and wound site (absent staining in sepsis 78% vs. 60% in controls). There were no significant differences in staining of PINP in intact skin or migrating edge or wound site between sepsis and control samples (Fig. S2).

## Discussion

Expression of ECM receptor syndecan‐1 and growth factors EGF, VEGF and TGF‐β was upregulated, while ECM component tenascin‐C and BM protein laminin‐332 were decreased in the blister wounds of septic patients compared to healthy controls. We did not find statistically significant differences in the amount of myofibroblast marker α‐SMA or ECM component PINP and BM protein type IV collagen in skin samples taken from septic patients and healthy controls.

### Expression of growth factors

There was significantly more EGF in both intact and migrating epithelium of septic patients than healthy controls. After acute skin injury, EGF is upregulated and is a major stimulator of skin re‐epithelialization and wound contraction by enhancing migration and proliferation of keratinocytes and fibroblasts [8, 25, 26, 27, 28, 29]. EGF receptor signalling suppresses inflammation in keratinocytes [30]; thus, the high levels of EGF in septic epithelium might be due to overwhelmed inflammatory response. It is suggested that extracellular vesicles are involved in forming the intestinal stem cell niche by transmitting EGF activity [31]. The role of extracellular vesicles in transporting growth factors in skin is yet to be investigated. We also found the expression of VEGF in epidermis and dermis to be upregulated in sepsis compared with healthy controls. Besides angiogenesis and lymphangiogenesis, VEGF has many other effects on the wound healing cascade, including inflammation, re‐epithelization and collagen deposition [32, 33]. On the contrary, in many studies overexpression of VEGF in healthy animals is ineffective in accelerating re‐epithelialization and wound closure [34]. Tissue hypoxia and inflammation lead to overexpression of VEGF in healing wounds [35, 36], which may explain the increased VEGF levels in septic skin. Increased vascular permeability, induced by VEGF, results in extravasation of plasma proteins, which is important for the formation of ECM.

In our study, a significantly stronger expression of TGF‐β was seen in the migrating basal epithelium in sepsis than in controls. In contrast, Sommer et al. [10] found TGF‐β levels to be decreased in the epidermis of septic mice. Generally, TGF‐β has increased expression in blister wounds in the early days after injury [37]. TGF‐β is a multifunctional growth factor that regulates re‐epithelialization, initiates inflammation by attracting inflammatory cells to the wound site, enhances synthesis and deposition of ECM components and reduces proteolytic degradation of ECM by downregulating protease synthesis [38, 39, 40]. TGF‐β is involved in upregulating VEGF expression and thus angiogenesis [41]. In several studies, overexpression of TGF‐β has induced skin inflammation, impairing wound healing [42]. Thus, the elevated levels of TGF‐β in septic skin might be a sign of a more severe cutaneous injury, hypoxia and inflammation, inhibiting re‐epithelialization and causing insufficient matrix composition in healing wounds.

We detected stronger expression of syndecan‐1 in all layers of intact and migrating wound edge epithelium in sepsis than in healthy skin. There are no studies about syndecan‐1 in wound healing in sepsis. Syndecan‐1 is a versatile cell‐surface proteoglycan induced in wound edge keratinocytes during tissue repair [43, 44]. Induction in the dermis may be associated with events that lead to inflammation and fibrosis [45]. Syndecan‐1 is a key component in regulating tissue regeneration and thus in maintenance of skin homeostasis. It is capable of binding to ECM proteins like tenascin and collagen, glycoproteins and growth factors (interleukins, VEGF and TGF‐β) [46]. Syndecan‐1 is also a cellular co‐receptor for laminin‐332, thus mediating keratinocyte migration during epidermal repair [47]. Many pathogens use syndecan‐1 as attachment receptors, and syndecan‐1 knockout mice display increased resistance to bacterial infections [48, 49]. Considering the wound repair process, the optimal balance of syndecan‐1 expression is unknown and related to the large number of growth factors regulating wound healing.

### Extracellular matrix and basement membrane

In our study, the manifestation of laminin‐332 was less in intact skin surrounding the superficial epidermal wound of septic patients than in healthy controls. Increased expression of laminin‐332 precedes that of other ECM components after an injury and successful re‐organization of BM enables re‐forming of the uniform epithelial surface [5]. Laminin‐332 ‐mediated cell signalling is suggested to play a central role in epidermal adhesion, cell survival, migration and regeneration [5, 50]. Disturbances in laminin‐332 expression in skin lead to dermal–epidermal separation, skin fragility and blistering [51]. All of these can be seen in septic patients.

In this study, the basal layer of migrating wound epithelium in sepsis had hardly any expression of tenascin‐c, in contrast to healthy control samples. In general, expression of tenascin is minor in normal uninjured skin, but intensifies near hyperproliferative epidermis and wound bed, the basal migrating keratinocytes being the main source of tenascin in the early phase of wound healing stimulated by TGF‐β [39, 52]. Tenascin is a multifunctional ECM protein that modulates cell adhesion, migration and proliferation, and it has a role in inflammatory and fibrotic processes in tissue repair [53]. Tenascin is also suggested to be a damage‐associated molecular pattern (DAMP) component [53, 54, 55]. Tenascin‐c expression has been used as an indicator of successful tissue repair [53]. In recent studies, the levels of tenascin in blood were upregulated in sepsis and associated with clinical severity, systemic inflammatory response and poor prognosis [54, 56]. Tenascin expression is suppressed by glucocorticoids in the granulation tissue of healing skin wounds in mice [57]. In our patient population, 47% had sepsis‐related anti‐inflammatory therapy with glucocorticoids, and this might also be one reason for the diminished amount of tenascin in septic skin. In conclusion, the decreased tenascin‐C in a septic skin wound probably diminishes keratinocyte and fibroblast migration and proliferation as well as interaction with cytokines and other ECM components, thus impairing the repair process.

Myofibroblast marker α‐SMA was almost absent in sepsis wounds compared with control wounds, but the result was at the limit of statistical significance. Myofibroblasts produce ECM components, and are also involved in contraction and closure of the wounds [58, 59]. Hypoxia reduces fibroblast proliferation and collagen formation in granulation tissue, which cause inadequate myofibroblast action and suppressed tissue contraction thus delaying wound closure and re‐epithelialization. [59]. In the present study, ECM proteins PINP and type IV collagen did not have significant changes in expression in wounded skin in sepsis patients compared with healthy controls. PINP is the precursor of type I collagen, and the amount of synthetized PINP is equal to that of type I collagen [60], which is the most common collagen in the body. Our group has previously demonstrated that in general PINP synthesis is quite unchanged but the degradation of type I collagen is enhanced in sepsis [21]. The levels of PINP are decreased in the fluid of early suction blisters in sepsis patients compared with healthy controls [18]. The results are contradictory, but the perspective is different. In the present study, we explore solid wounded tissue, whereas in our earlier studies the amount of PINP has been quantified from blood and blister fluid, which resembles interstitial fluid. It might have been more informative to measure procollagen type III aminoterminal propeptide, the precursor of type III collagen. The fast appearance of type III collagen in healing wound is associated with an early increase in collagen synthesis [61]. In our previous study, the expression of type IV collagen in inviolable skin was decreased in sepsis on Day 1 compared to healthy controls [17], but we could not detect any significant differences between the groups on Day 3 in wounded skin.

Taken together, although growth factor and syndecan expression was shown to be increased, ECM and BM protein expression was depressed, as markers of dysregulated wound healing in sepsis. In our previous in vitro study, we found lower levels of EGF in sepsis sera, and keratinocyte monolayer wounds treated with sepsis sera had decreased keratinocyte migration and proliferation compared with healthy sera. Adding a small amount of EGF to sepsis or healthy sera enhanced human keratinocyte migration significantly [8]. The promigratory effect of supplemented EGF was limited to 10 ng/ml or less. Cell migration was also suppressed by blocking EGF receptor (EGFR) [8]. EGF or EGFR knockout mice showed impaired wound re‐epithelialization [62, 63], and EGF levels were also decreased in chronic human wounds [64]. Furthermore, VEGF concentrations in serum have been shown to be increased in patients with sepsis [8, 65]. Our findings and other studies suggest resistance to growth factor stimulus at the cellular level in sepsis. There are many elements that restrict growth factor influence in sepsis, such as wound contamination and biofilm formation, increased levels of matrix metalloproteinases and decreased tissue inhibitor of metalloproteinases, dysregulated synthesis of other growth factors and ECM components, oedema, hypoxia and use of corticosteroids [2, 7, 27, 39, 57].

To our knowledge, this is the first human study reporting immunohistochemical alterations in skin wounds of septic patients. However, the study has some limitations. Although the total number of samples was 190 (106 sepsis and 84 control samples), it is still statistically challenging. Our samples cover days 3 to 7, which is the overlapping period of inflammatory and proliferative phases [27, 58]. Unfortunately, we were able to obtain only 2 to 4 samples per day from septic patients due to consent or other limiting issues. Thus, we could not compare the alterations between different days in the tissue repair process. Our results as an observational data should be considered hypothesis‐generating for new insights into wound healing in sepsis. There are numerous other molecules and components besides these ones we analysed that participate in wound healing. More studies are needed to gain a deeper understanding of tissue repair in sepsis.

## Conclusions

In sepsis, growth factor and syndecan expression was enhanced, while extracellular matrix and basement membrane protein were depressed. The results emphasize dysregulated skin wound healing and abnormal interplay of growth factors and ECM in sepsis in sepsis.

## Funding information

This study was supported by grants from the Medical Research Center of Oulu and University of Oulu Graduate School, the Oulu University Hospital VTR fund, the Finnish Cultural Foundation, the Lapland Regional Fund and the Finnish Medical Foundation.

## CONFLICT OF INTERESTS

The authors have no competing interests to declare.

## ETHICAL APPROVAL

The study protocol (register number 50/2005) was approved by the Regional Ethics Committee of the Northern Ostrobothnia Hospital District, Finland. All patients or their next of kin gave written consent for the study.

## AUTHOR CONTRIBUTIONS

All authors participated in the study design. MK and FG collected the data and samples. HJ, MK and TS analysed the samples by microscope. HJ performed the statistical analyses. HJ drafted the manuscript with VK, TS and TA. All authors read and approved the final manuscript.

## Supporting information


Supplementary Material.

## Data Availability

The data sets generated and/or analysed during the current study are available from the corresponding author on reasonable request.
