# Supplementary material for: Growth factor expression is enhanced, and extracellular matrix proteins are depressed in healing skin wounds in septic patients compared with healthy controls
Source: APMIS. 2022 Jan 23;130(3):155–68. doi: 10.1111/apm.13175 (PMC9305760; doi:10.1111/apm.13175)
Supplement: Supplementary file 1 — Supplementary Material. [file APM-130-155-s001.docx]

Supplementary material

**Growth factor expression is enhanced, and ECM proteins are depressed in healing wounds in septic patients compared with healthy controls**

Henna Jaurila^* 1 2^, Marjo Koskela^1^, Vesa Koivukangas^1^, Fiia Gäddnäs^1^, Tuula Salo^† 2 3^, Tero I Ala-Kokko^† 1^

¹ Research Group of Surgery, Anesthesia and Intensive Care, Oulu University Hospital, Oulu, Finland, Medical Research Center Oulu, University of Oulu, Finland

^2^ Cancer and Translational Medicine Research Unit, Faculty of Medicine, Medical Research Center Oulu, University of Oulu, Finland

^3^ Research Group of Oral Health Sciences, Oulu University Hospital, Medical Research Center Oulu, University of Oulu, Finland

* Correspondence: henna.jaurila@fimnet.fi

^†^ With equal contribution

Supplementary Table S1. Antibodies used in the immunohistochemical staining.

| Antigen | Type of antibody | Source of antibody | Dilution | Detection kit |
| --- | --- | --- | --- | --- |
| Alpha-sma | mouse monoclonal, clone 1A4 | DakoCytomation Denmark A/S, Glostrup, Denmark | 1:1000 | EnVision Detection Systems, DakoCytomation Denmark A/S, Glostrup, Denmark |
| CD138 =syndecan-1 | mouse monoclonal, clone 5F7 | ThermoScientific, Thermo Fisher Scientific, Waltham, MA 02451, USA | 1:40 | EnVision Detection Systems, Dako |
| EGF | goat polyclonal, AF236 | R&D systems, Inc, Minneapolis, MN, USA | 1:100 | Biocare Goat HRP-Polymer Kit, Biocare Medical, LLC, Concord, CA, USA |
| Laminin-332 | rabbit polyclonal | Dako, Glostrup, Denmark | 1:400 | UltraVision Large Volume Detection System/LabVision, HRP, Thermo Fisher Scientific Inc. Fremont, CA, USA |
| PINP | rabbit polyclonal | A kind gift from Prof. of Clin Chem Juha Risteli, University of Oulu, Oulu, Finland [1] | 1:5000 | EnVision Detection Systems, Dako |
| TGF-beta | rabbit polyclonal, RB-9262-P1 | NeoMarkers, Fremont, CA, USA | 1:100 | EnVision Detection Systems, Dako |
| Tenascin | mouse monoclonal, NCL-TENAS-C | NovoCastra Laboratories Ltd., Newcastle upon Tyne, UK | 1:150 | EnVision Detection Systems, Dako |
| Type IV collagen | mouse monoclonal, clone CIV 22 | Dako, Glostrup, Denmark | 1:100 | UltraVision Large Volume Detection System/LabVision |
| VEGF | mouse monoclonal, A-20 | SantaCruz Biotechnology, Santa Cruz, CA, USA | 1:400 | EnVision Detection Systems, Dako |

1. Risteli J, Elomaa I, Niemi S, Novamo A, Risteli L. Radioimmunoassay for the pyridinoline cross-linked carboxy-terminal telopeptide of type I collagen: A new serum marker of bone collagen degradation. Clin Chem. 1993;39:635–40.

**Supplementary Fig. S1.** Immunohistochemical staining of type IV collagen in skin samples of septic patients (A) and healthy controls (B) on day 6 post wounding. No significant staining differences are seen between the two groups. Staining intensity is reported as absent (-), mild (+), moderate (++) or strong (+++), and the percentages are shown (C). The bar at the bottom of the figure is equal to 100 µm. Statistical significances between study groups are indicated by brackets and significant P-values (P<0.05) are marked with an asterisk

A B


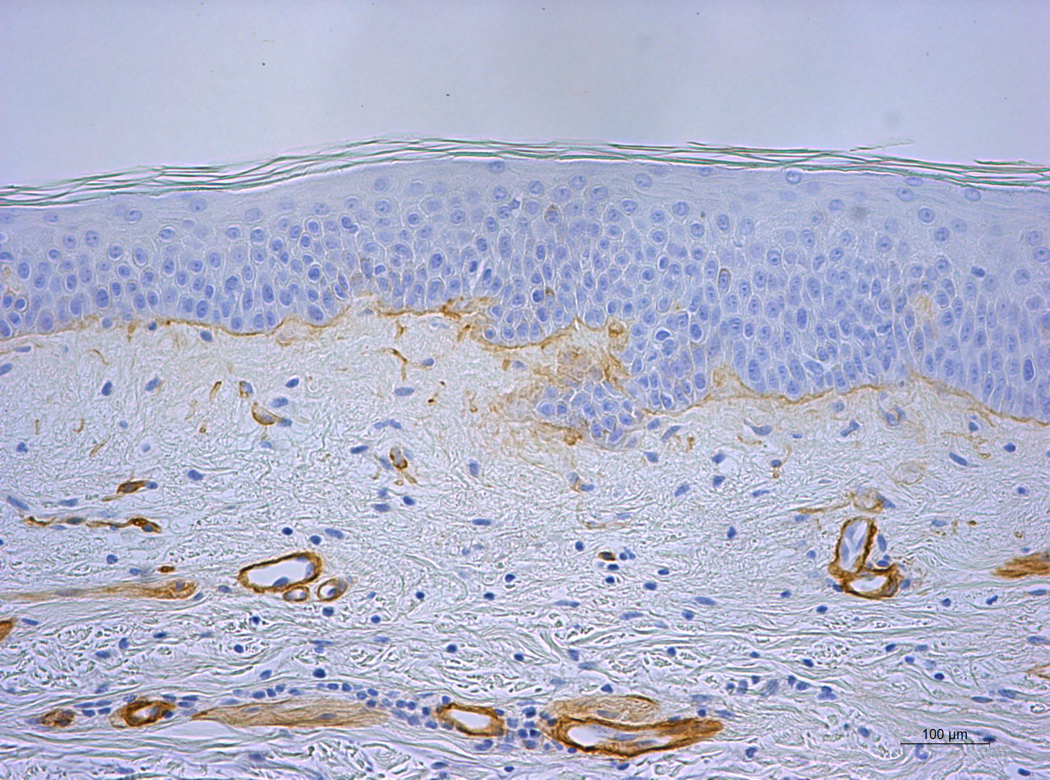

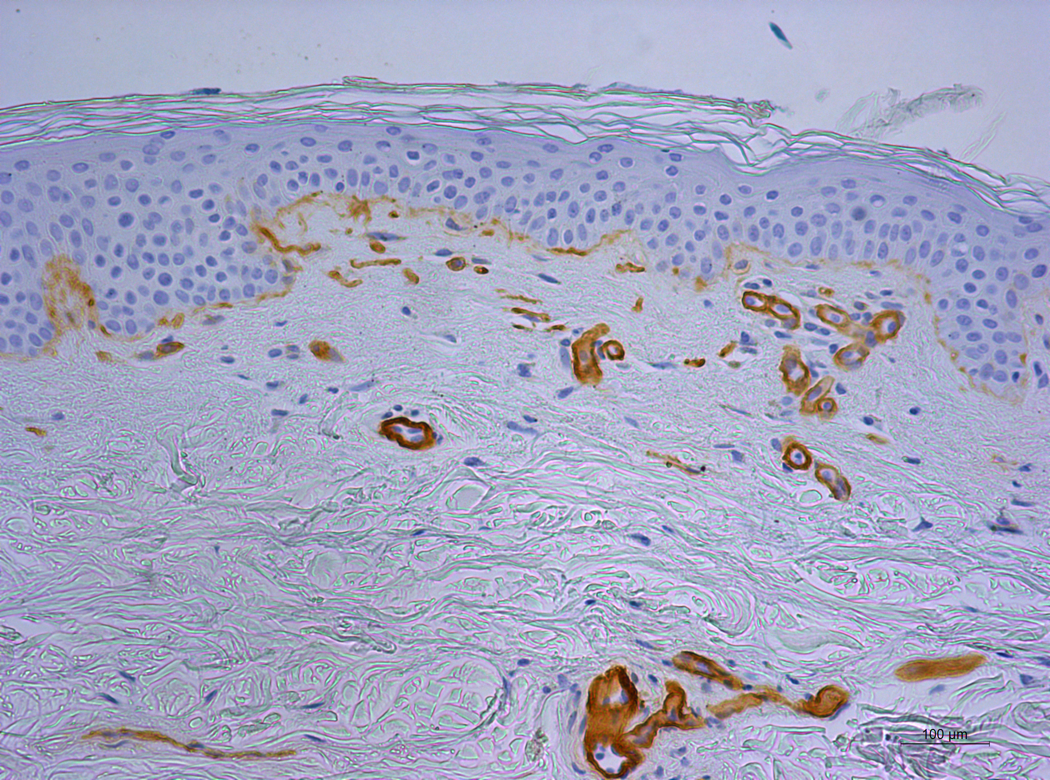


C

**Supplementary Fig. S2** Immunohistochemical staining of procollagen type I aminoterminal propeptide (PINP) in skin on day 4 post wounding. Samples of septic patients (A) and healthy controls (B) are shown. Staining of samples is similar in the two groups. Staining intensity is reported as absent (-), mild (+), moderate (++) or strong (+++), and the percentages are shown (C). The bar at the bottom of the figure is equal to 100 µm. Statistical significances between study groups are indicated by brackets and significant P-values (P<0.05) are marked with an asterisk

A B


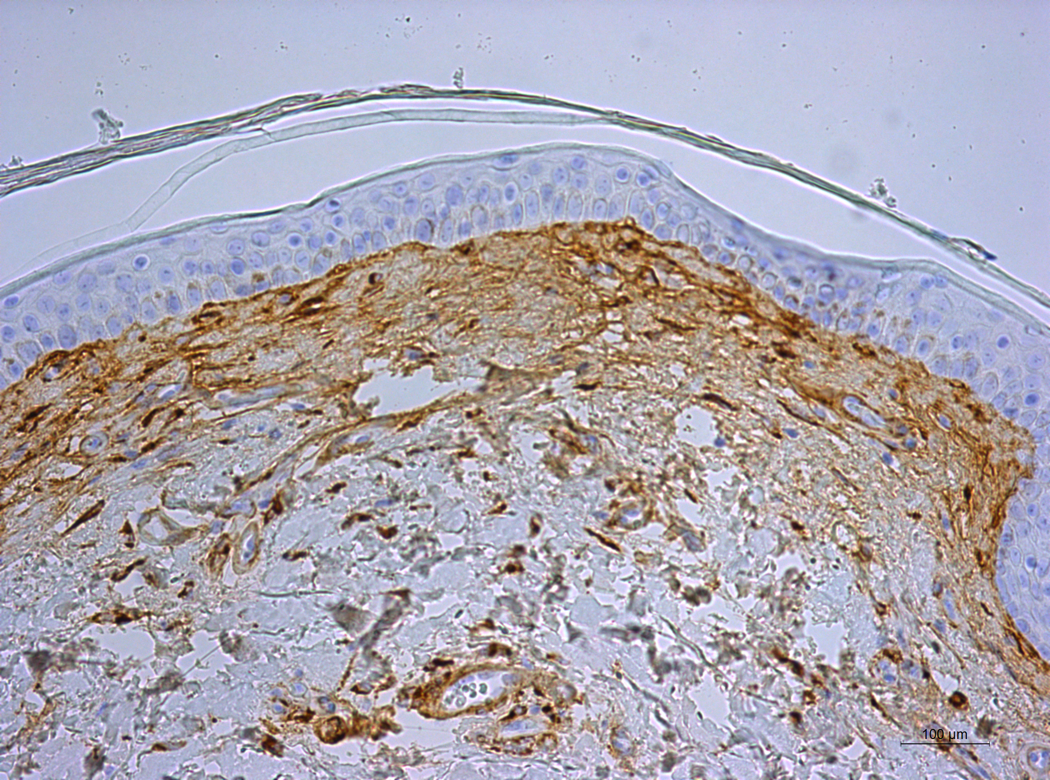

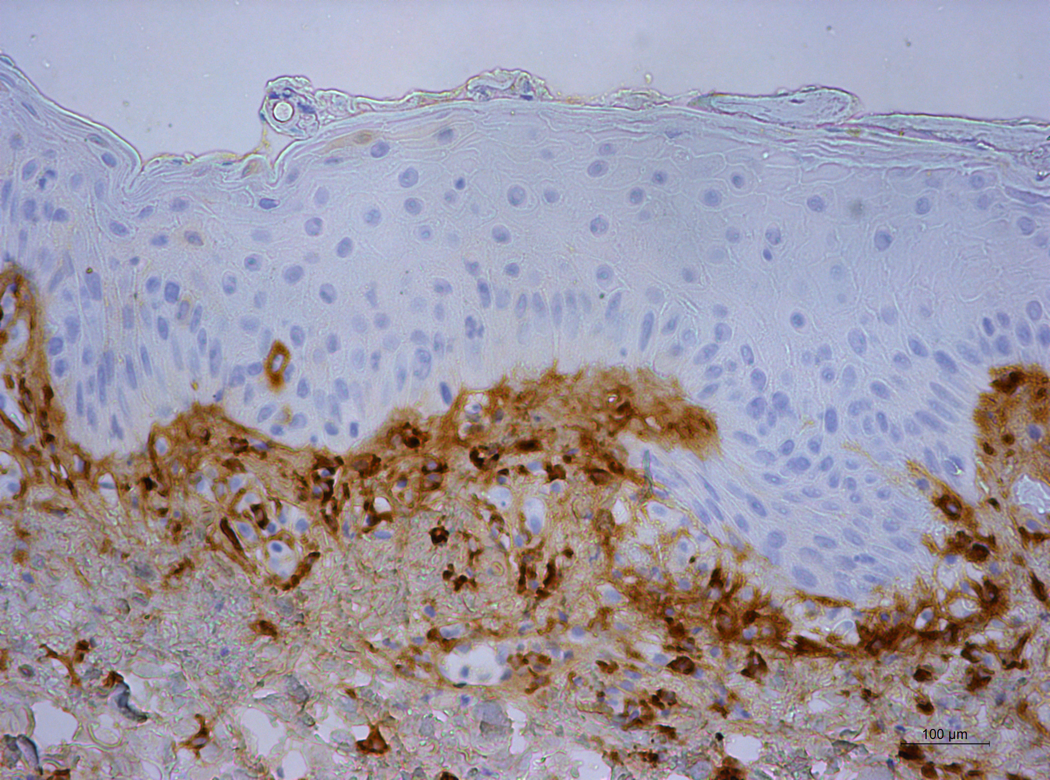


C
